# Predictable Phenotypes of Antibiotic Resistance Mutations

**DOI:** 10.1128/mBio.00770-18

**Published:** 2018-05-15

**Authors:** M. Knopp, D. I. Andersson

**Affiliations:** a Department of Medical Biochemistry and Microbiology, Uppsala University, Uppsala, Sweden; McMaster University

**Keywords:** antibiotic resistance, epistasis, fitness, Salmonella, Escherichia coli, Strain specificity

## Abstract

Antibiotic-resistant bacteria represent a major threat to our ability to treat bacterial infections. Two factors that determine the evolutionary success of antibiotic resistance mutations are their impact on resistance level and the fitness cost. Recent studies suggest that resistance mutations commonly show epistatic interactions, which would complicate predictions of their stability in bacterial populations. We analyzed 13 different chromosomal resistance mutations and 10 host strains of Salmonella enterica and Escherichia coli to address two main questions. (i) Are there epistatic interactions between different chromosomal resistance mutations? (ii) How does the strain background and genetic distance influence the effect of chromosomal resistance mutations on resistance and fitness? Our results show that the effects of combined resistance mutations on resistance and fitness are largely predictable and that epistasis remains rare even when up to four mutations were combined. Furthermore, a majority of the mutations, especially target alteration mutations, demonstrate strain-independent phenotypes across different species. This study extends our understanding of epistasis among resistance mutations and shows that interactions between different resistance mutations are often predictable from the characteristics of the individual mutations.

## IMPORTANCE

The spread of antibiotic-resistant bacteria imposes an urgent threat to public health. The ability to forecast the evolutionary success of resistant mutants would help to combat dissemination of antibiotic resistance. Previous studies have shown that the phenotypic effects (fitness and resistance level) of resistance mutations can vary substantially depending on the genetic context in which they occur. We conducted a broad screen using many different resistance mutations and host strains to identify potential epistatic interactions between various types of resistance mutations and to determine the effect of strain background on resistance phenotypes. Combinations of several different mutations showed a large amount of phenotypic predictability, and the majority of the mutations displayed strain-independent phenotypes. However, we also identified a few outliers from these patterns, illustrating that the choice of host organism can be critically important when studying antibiotic resistance mutations.

## INTRODUCTION

The evolution and dissemination of antibiotic resistance are threats to many advances in modern medicine, and infections caused by resistant bacteria impose a considerable health and economic burden (1). To efficiently combat this trend, we need a deeper understanding of the factors that contribute to resistance evolution to allow the development of methods to accurately predict the emergence and spread of resistance mutations/genes, which could potentially influence treatment regimens, surveillance, and drug design.

A key factor that influences the evolutionary success of any type of resistance mechanism is its associated fitness effect. Chromosomal resistance mutations often have a negative impact on the host bacterium (2–4), and these deleterious effects are often associated with a reduction in the bacterial growth rate. For example, mutations in target genes such as *rpoB* (5, 6), *rpsL* (7), *fusA* (8), or *fmt* (9) can reduce the efficiency of the affected process. Other mutations that are not altering the target of an antibiotic can also negatively affect the fitness of a bacterium. For example, mutations that alter the channel properties or levels of expression of outer membrane proteins can reduce the influx of antibiotics through the outer membrane (10) but they can also reduce uptake of other, beneficial compounds, including nutrients, which can consequently cause a reduced growth rate of porin-deficient mutants (11, 12). Activation of efflux pumps on the other hand can impose a metabolic burden by exporting beneficial compounds out of the cell (13–17).

Considering the fitness costs that accompany many chromosomal resistance mutations, it is expected that resistant bacteria are outcompeted by susceptible high-fitness wild-type strains in the absence of antibiotics. However, resistant mutants can acquire second-site mutations that ameliorate the fitness burden and restore growth rates to wild-type levels (12, 18–22). While these compensatory mutations increase the fitness in a bacterium carrying the resistance mutation, they can also be deleterious if they appear in a susceptible genetic background (23). Recent studies that show that chromosomal resistance mutations can interact epistatically to reduce the overall fitness cost are relevant in this context (24–30). These epistatic interactions hamper our ability to predict the emergence and evolutionary success of resistance mutations. Furthermore, as the phenotypic effect of resistance mutations can depend on the presence of second-site mutations, it raises the question of how much influence the genetic background has that the mutations appear in. Most research is done in laboratory strains, and the insights generated, for example on specific resistance mutations, are often assumed to be transferable to other strains or even species, but if epistatic interactions are prevalent, this assumption is questionable (31).

**FIG 1:**
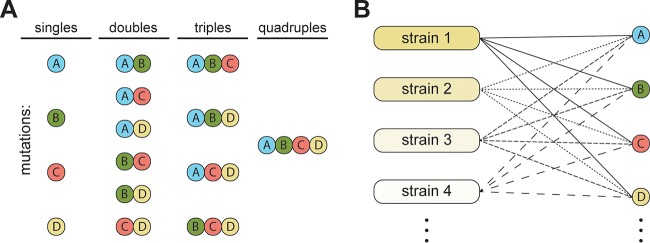
Experimental setup. (A) Chromosomal resistance mutations were used to construct sets of single, double, triple, and quadruple combinatorial mutants in *S*. Typhimurium LT2, and the effects on growth and resistance were examined. (B) A set of 13 mutations were individually introduced into 10 different genetic backgrounds (generating 130 strains) to study how the genetic background of the strain affects the phenotypic expression of the resistance mutations.

Here, we present an extensive study on the epistatic interactions of chromosomal resistance mutations. We investigated the dependence of the phenotypic effect of selected resistance mutations (i) on the presence of other resistance mutations by constructing sets of combinatorial mutants in Salmonella enterica serovar Typhimurium strain LT2 (hereafter referred to as *S*. Typhimurium LT2) and (ii) in different genetic contexts by studying the resistance mutations in a wide set of strains with diverse genetic distances consisting of nine different strains of *Salmonella* and Escherichia coli K-12 MG1655 (hereafter referred to as E. coli MG1655) (Fig. 1).

## RESULTS

### Chromosomal resistance mutations studied.

We investigated 13 different chromosomal resistance mutations which were subsequently characterized for their epistatic robustness (Table 1). These genes were previously identified as common mutational targets in *in vitro* selections for antibiotic resistance and represent a broad spectrum of chromosomal resistance mutations, a majority of which have also been linked to resistance development in clinical strains. The set of genes encodes proteins involved in a wide variety of physiological functions ranging from central cellular processes (for example, *gyrA* for replication, *rpoB* for transcription, and *rpsL*, *fusA*, *fmt*, and *gidB* for translation) to regulatory proteins affecting global (*ompR* and *cysB*) or local (*acrR*) gene expression. Six mutations constitute target alterations that generally confer a high increase in resistance to a specific antibiotic/antibiotic class. Mutations in the other seven genes mostly affect the import or export of antibiotics by downregulation of porins (*ompR* and *lon*), remodeling of the cell envelope (*mgrB* and *pmrA*), or activation of efflux pumps (*marR* and *acrR*). This group of mutations generally provides only small increases in resistance but resistance to a wide range of antibiotics. All mutations were constructed in *S*. Typhimurium LT2, and the whole genome of each strain was subsequently sequenced to confirm the presence of the desired mutations and the absence of off-target mutations. The resulting 13 mutants served as starting points for studies of combinatorial epistatic effects as well as the strain dependence of these mutations.

**TABLE 1:** Chromosomal resistance mutations

Gene	Gene function^a^	Mutant allele	Fitness cost (%)^a,b^	Resistance to the following^a^:	Resistance mechanism	Reference(s)
*gyrA^c^*	Gyrase	S83F	BDL	CIP	Target alteration	51
*rpoB^c^*	RNA PM	S531L	23	RIF	Target alteration	52
*rpsL^c^*	Ribosomal protein	K42N	22	STM	Target alteration	53
*fusA^c^*	EF-G	P413L	52	FUS	Target alteration	54
*fmt^c^*	Formyltransferase	T12R	67	ACT	Target bypass	55
*gidB*	Methyltransferase	Q167*	BDL	STM	Target alteration	56
*ompR^d^*	TF	Δ	BDL	MDR	Influx/efflux	57, 58
*marR^d^*	TF	Q110*	BDL	MDR	Influx/efflux	59
*lon^d^*	Protease	Q137*	43	MDR	Influx/efflux	60, 61
*mgrB^d^*	Autoregulator	Δ	BDL	Col	Influx/efflux	62
*pmrA*	TF	G53E	BDL	Col	Influx/efflux	63
*acrR*	TF	H115Y	BDL	MDR	Influx/efflux	64
*cysB*	TF	Δ	26	MEC	Unknown	65

a Abbreviations: BDL, below detection limit (<3%); PM, polymerase; EF, elongation factor; TF, transcription factor; CIP, ciprofloxacin; RIF, rifampin; STM, streptomycin; FUS, fusidic acid; ACT, actinonin; Col, colistin; MEC, amdinocillin; MDR, multidrug resistance.

b The value represents the fitness cost of the mutation in *S*. Typhimurium LT2, expressed as the relative exponential growth rate compared to the growth rate of the wild type.

c These mutations were used to construct combinatorial sets of target alteration mutations.

d These mutations were used to construct combinatorial sets of regulator mutations.

### Epistatic interactions between resistance mutations.

**FIG 2:**
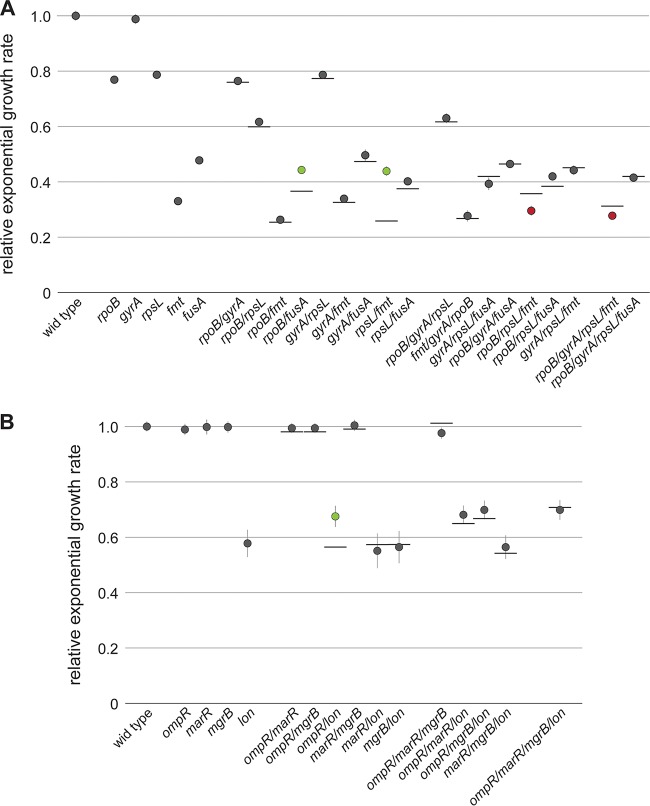
Relative growth rates of combinatorial mutants in *S*. Typhimurium LT2. (A) Combinations of target alteration mutations *rpoB* S531L, *gyrA* S83F, *rpsL* K42N, *fmt* T12R, and *fusA* P413L. (B) Combinations of regulator mutations Δ*ompR*, *marR* Q110*, Δ*mgrB*, and *acrR* H115Y. Values are the means of a minimum of four biological replicates and two technical replicates. Error bars represent standard deviations. The horizontal lines indicate the expected growth rates if no epistatic interaction is present. Green and red circles represent positive and negative epistatic interactions, respectively.

From the set of resistance alleles (Table 1), we selected five genes that cause resistance by target alteration (*gyrA*, *fusA*, *rpoB*, *rpsL*, and *fmt*) to investigate whether the phenotype of these mutants is dependent on the presence or absence of other target alteration mutations. These five genes were chosen to represent resistance mechanisms to different classes of antibiotics that target different cellular processes. We constructed all possible single, double, triple, and quadruple mutants in *S*. Typhimurium LT2. Combining the mutant *fusA* and *fmt* alleles failed after numerous attempts, indicating a lethal phenotype of this specific combination. We then selected four representative mutant alleles of genes encoding regulators (*ompR*, *marR*, *lon*, and *mgrB*) that were previously selected for increased resistance to different antibiotics. We constructed all possible single, double, triple, and quadruple mutants, generating a set of 38 mutants, and the whole genomes of all of these mutants were sequenced to ascertain that no off-target mutations were inadvertently introduced during strain construction. All strains with off-target mutations were reconstructed to generate a clean set of mutants. The resulting mutants were then subjected to phenotypic characterization. Relative fitness is often measured by competition experiments, either in animals or in laboratory media, between a susceptible and resistant strain. This is generally superior to fitness estimates based on only exponential growth. However, since by design, competition experiments cannot be performed in monoculture, the fitness of a strain will be influenced by the competitor, for example via secretion of detrimental compounds, depletion of nutrients, or changes in pH. This phenomenon was observed in a study by de Sousa et al. (32), where two mutants showed similar growth defects in competition with a wild-type strain, but in a direct competition between the mutant strains, one mutation was highly detrimental. To avoid this effect, we used exponential growth rate determinations, as they can be performed in monoculture and allow the analysis of isolated traits without interference caused by the competitor. Target alteration mutations had much stronger impact on fitness, with *rpoB* S531L, *rpsL* K42N, *fusA* P413L, and *fmt* T12R reducing exponential growth rate by 23%, 22%, 52%, and 67%, respectively (Fig. 2). Only *gyrA* S83F did not affect the growth rate within our detection limit (3% for growth rate determinations using the Bioscreen C reader). Among the regulator mutants, neither Δ*ompR*, *marR* Q110*, nor Δ*mgrB* affected growth rates. Only *lon* Q137* caused a severe growth reduction by 43%. In addition, all *S*. Typhimurium LT2 mutants carrying *lon* Q137* exhibited a mucoid phenotype, which is a known characteristic of *lon* mutants in *Salmonella* (33). The level of mucoidy could also influence the determination of exponential growth rate using the Bioscreen C reader, since cells attached to the plastic will not be measured. Thus, reduced growth in *lon* mutants might be partly due to biofilm formation.

We observed a very good predictability of growth rates among the combinatorial mutants for target alteration and regulator mutations, with 24 of 29 mutants showing growth rates similar to the predicted additive values (“additive” phenotypes were calculated by multiplication of the effects by single mutants). Only three pairs of all double mutants deviated significantly. The strongest epistatic effect was observed for the combination of *rpsL* K42N with *fmt* T12R where the growth rate was observed to be more than 70% higher than the predicted additive value (relative growth rate of 0.44 compared to 0.26). In this pair, the deleterious *rpsL* K42N mutation, which by itself reduces the growth rate by 22%, acts as a compensatory mutation in an *fmt* T12R background, increasing the relative growth rate from 0.33 to 0.44, exhibiting a clear sign of epistasis. The only other case of sign epistasis was observed for the combination of Δ*ompR* and *lon* Q137*. Despite Δ*ompR* not causing a detected growth defect in our assay, it functioned as a compensatory mutation in a *lon* Q137* background, increasing relative growth from 0.57 to 0.67. A smaller epistatic interaction was observed for the combination of *rpoB* S531L and *fusA* P413L (relative growth of 0.44 instead of 0.36). Among the triple and quadruple mutants, we could not detect any case of positive epistasis; however, we found that the combination of *rpoB* S531L, *rpsL* K42N, and *fmt* T12R showed a 16% lower growth rate and the quadruple mutant with *rpoB* S531L, *gyrA* S83F, *rpsL* K42N, and *fmt* T12R showed an 11% lower growth rate compared to the theoretical additive value.

We also investigated possible epistatic effects of these mutations on resistance levels. In each combinatorial set, we tested all strains for the antibiotic that the single mutants were initially selected on. For target alteration mutations, we tested rifampin, ciprofloxacin, streptomycin, fusidic acid, and actinonin, and for regulator mutations, we tested ciprofloxacin, tigecycline, ertapenem, and colistin (see Fig. S1 and S2 in the supplemental material). Only mutants carrying *fmt* T12R showed MIC values deviating from the predicted additive values. These mutants had slightly decreased MICs of ciprofloxacin, rifampin, and streptomycin and an increased MIC of fusidic acid. Notably, these mutants also had the largest growth defects. For all other mutants, we did not observe any epistatic effects on the resistance phenotype. Only minor deviations (approximately twofold) were observed from predicted MICs, which is within the margin of error for Etest determinations.

10.1128/mBio.00770-18.1 FIG S1  MICs of different antibiotics with regulator mutation combinations. MICs were determined using Etest strips on Mueller-Hinton (MH) agar. Values represent the fold change compared to the MIC of wild-type *S*. Typhimurium LT2. Download FIG S1, PDF file, 0.1 MB. Copyright © 2018 Knopp and Andersson. 2018 Knopp and Andersson This content is distributed under the terms of the Creative Commons Attribution 4.0 International license.

10.1128/mBio.00770-18.2 FIG S2  MICs of target mutation combinations. MICs were determined using Etest strips on MH agar (nalidixic acid, streptomycin, and fusidic acid) or microdilution (actinonin and rifampin). Values represent the fold change compared to the MIC of wild-type *S*. Typhimurium LT2. Download FIG S2, PDF file, 0.1 MB. Copyright © 2018 Knopp and Andersson. 2018 Knopp and Andersson This content is distributed under the terms of the Creative Commons Attribution 4.0 International license.

### Selected host strains with differing genetic distances.

**FIG 3:**
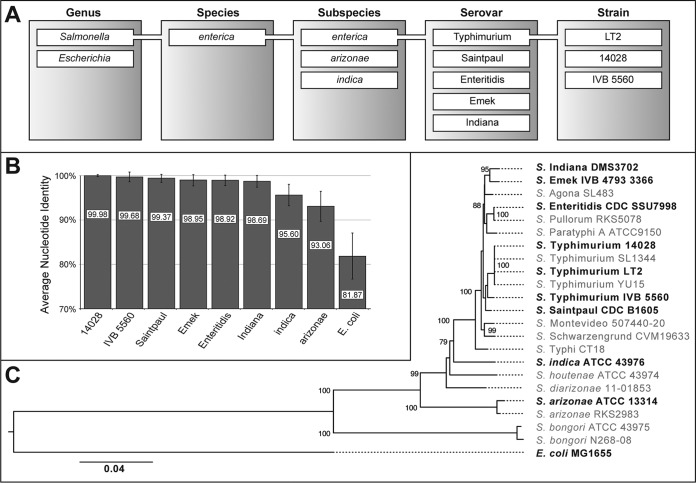
Phylogenetic relationships between selected host strains. (A) The host strain used in this study include representatives from different strains, serovars, subspecies, and genera to represent a broad spectrum of genetic backgrounds. (B) Average nucleotide identities (ANI) of all host strains versus *S*. Typhimurium LT2 range from 99.98% to 81.87%. For a complete comparison of ANI between all strains, see Table S4 in the supplemental material. (C) Maximum likelihood tree of the strains used in this study supplemented with other representatives of the *Salmonella* clade (rooted with E. coli). The tree was inferred from seven housekeeping genes (*hisD*, *purE*, *sucA*, *thrA*, *aroC*, *dnaN*, and *hemD*). Numbers on the branches represent the support values of 1,000 bootstrap replicates. Only values of >70 are presented. The bar indicates the number of substitutions per site.

To further investigate the epistatic robustness of chromosomal resistance mutations, we studied the phenotypic expression of 13 resistance mutations (Table 1) in 10 different strain backgrounds with differing genetic distances (Fig. 3A). The selected hosts spanned strains within the same serovar sharing an average nucleotide identity (ANI) of 99.98% (*S*. Typhimurium LT2 versus *S*. Typhimurium 14028) to members of different subspecies and genera with ANIs of as little as 81.87% (*S*. Typhimurium LT2 versus E. coli MG1655) as shown in Fig. 3B (for clarity, shortened strain designations are used throughout the text, but complete designations are listed in Table S1 in the supplemental material). The relationship between all host strains is shown in a phylogenetic tree based on multilocus sequence typing of the seven housekeeping genes *hisD*, *purE*, *sucA*, *thrA*, *aroC*, *dnaN*, and *hemD* (Fig. 3C). The exponential growth rates of the host strains compared to *S*. Typhimurium LT2 ranged between 1.06 (*S*. Enteritidis) and 0.89 (S. indica) (Fig. S3). All 13 mutations were constructed in each of the 10 host strains generating 130 individual mutants (Table S1). Three hosts (*S*. Typhimurium LT2, *S*. Saintpaul, and E. coli MG1655) were manipulated using lambda red recombineering. The resulting *S*. Typhimurium LT2 mutants were then used as a donor for P22 transductions into the remaining seven strains. Two of these (S. enterica subsp. indica and S. enterica subsp. arizonae) are naturally P22 resistant, most likely due to changes in the O antigen which serves as a receptor for P22. To introduce P22 susceptibility, we transformed these strains with a cosmid (pPR1347) that carries the *rfb* and *rfc* gene cluster from *S*. Typhimurium LT2 (34). As long as cosmid selection was maintained, the cells showed P22 sensitivity. After removal of selective pressure, the cosmid and with it P22 sensitivity was rapidly lost. The complete workflow of the strain construction is summarized in Fig. S4 and S5. In contrast to lambda red recombineering, which is a highly precise genetic tool to introduce specific genetic changes, P22 transduction results in recombination of larger DNA fragments of up to 43,500 bp, with the average size of a recombining fragment being estimated to be around 10,000 bp (35). Transduction between different strains can result in cotransduction of sequence differences. To estimate the size of the recombining fragment, we sequenced the whole genomes of eight transductants (Table S2).

10.1128/mBio.00770-18.3 FIG S3  Relative exponential growth rates of host strains. The values represent the growth rates of each wild-type strain relative to the exponential growth rate of *S*. Typhimurium LT2. Error bars represent the standard deviations of a minimum of four biological replicates and two technical replicates. Download FIG S3, PDF file, 0.1 MB. Copyright © 2018 Knopp and Andersson. 2018 Knopp and Andersson This content is distributed under the terms of the Creative Commons Attribution 4.0 International license.

10.1128/mBio.00770-18.4 FIG S4  Workflow for construction of mutant alleles of essential genes. Strains with target alteration mutations in *rpoB*, *gyrA*, *rpsL*, *fusA*, and *fmt* were used as donor lysates and transduced into *S*. Typhimurium LT2. The resulting mutants were used to introduce forced duplication containing the gene of interest for subsequent transduction into *S*. Typhimurium 14028, *S*. Typhimurium IVB 5560, *S*. Emek, *S*. Enteritidis, *S*. Indiana, *S. enterica* subsp. *indica*, and *S. enterica* subsp. *arizonae*. In the case of *S*. Saintpaul and E. coli MG1655, the mutations were introduced by lambda red recombineering. Abbreviations: ATB, antibiotic; dsLR, double-stranded lambda red; cam, chloramphenicol; kan, kanamycin; ssLR, single-stranded lambda red; fDup, forced duplication. Download FIG S4, PDF file, 0.1 MB. Copyright © 2018 Knopp and Andersson. 2018 Knopp and Andersson This content is distributed under the terms of the Creative Commons Attribution 4.0 International license.

10.1128/mBio.00770-18.5 FIG S5  Workflow for construction of mutant alleles of nonessential genes. A *cat*-*sacB* cassette was introduced in the gene of interest (*gidB*, *marR*, *acrR*, *ompR*, *mgrB*, *cysB*, *lon*, and *pmrA*) in *S*. Typhimurium LT2, *S*. Saintpaul, and E. coli MG1655 and subsequently removed by single-stranded lambda red recombineering with oligonucleotides carrying the desired mutation. The *S*. Typhimurium LT2 mutants were then used to introduce forced duplication spanning the mutations which were subsequently transduced into other target strains. Abbreviations: ATB, antibiotic; dsLR, double-stranded lambda red; cam, chloramphenicol; kan, kanamycin; ssLR, single-stranded lambda red; fDup, forced duplication; goi, gene of interest. Download FIG S5, PDF file, 0.1 MB. Copyright © 2018 Knopp and Andersson. 2018 Knopp and Andersson This content is distributed under the terms of the Creative Commons Attribution 4.0 International license.

10.1128/mBio.00770-18.6 TABLE S1  Host strains used in this study. Download TABLE S1, DOCX file, 0.05 MB. Copyright © 2018 Knopp and Andersson. 2018 Knopp and Andersson This content is distributed under the terms of the Creative Commons Attribution 4.0 International license.

10.1128/mBio.00770-18.7 TABLE S2  Characteristics of P22 transducing fragments. Sizes were estimated by aligning the reads to a reference LT2 genome. The distance to the first strain-specific single nucleotide polymorphism (SNP) up- and downstream of the transduced resistance mutation gives the maximum size of the transduced fragment. From a mapping of the wild-type sequence to LT2, the closest SNP from each (which was replaced in the transductant with the LT2-specific sequence) gives the minimal transducing fragment size. Download TABLE S2, DOCX file, 0.04 MB. Copyright © 2018 Knopp and Andersson. 2018 Knopp and Andersson This content is distributed under the terms of the Creative Commons Attribution 4.0 International license.

### Strain-specific effects of target alteration mutations.

**FIG 4:**
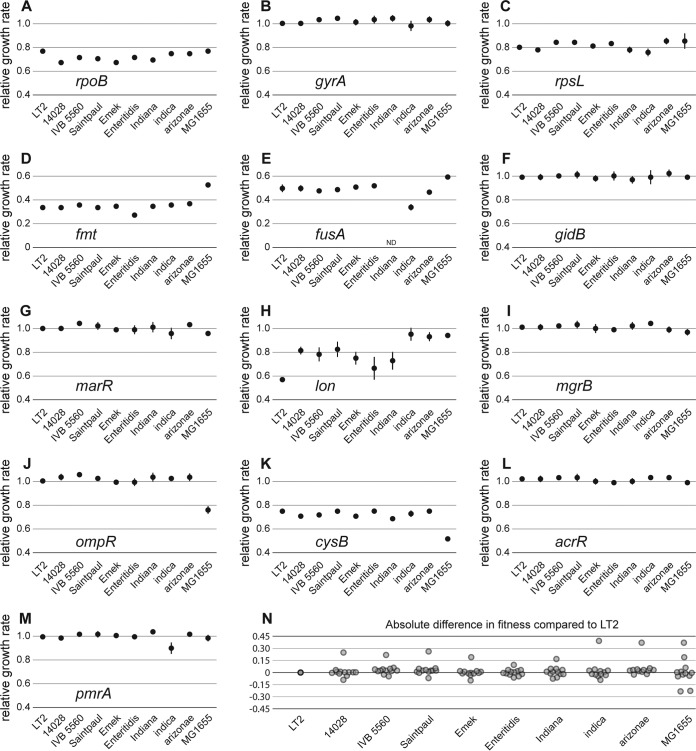
Effects of mutant alleles on exponential growth rates in different genetic backgrounds. (A to M) Exponential growth rates of 10 strains with differing genetic distances carrying the indicated mutant allele relative to the respective wild-type strain. All values represent the means of a minimum of four biological replicates and two technical replicates with error bars representing the standard deviations. (N) Absolute fitness difference (as a percentage) of all mutant constructs compared to the corresponding mutants in *S*. Typhimurium LT2. The values were obtained by subtracting the relative growth rate of a mutant in any given strain with the relative growth rate of *S*. Typhimurium LT2 carrying the identical mutation. For complete strain designations and phylogenetic relationships, see Fig. 3 and Table S1.

**FIG 5:**
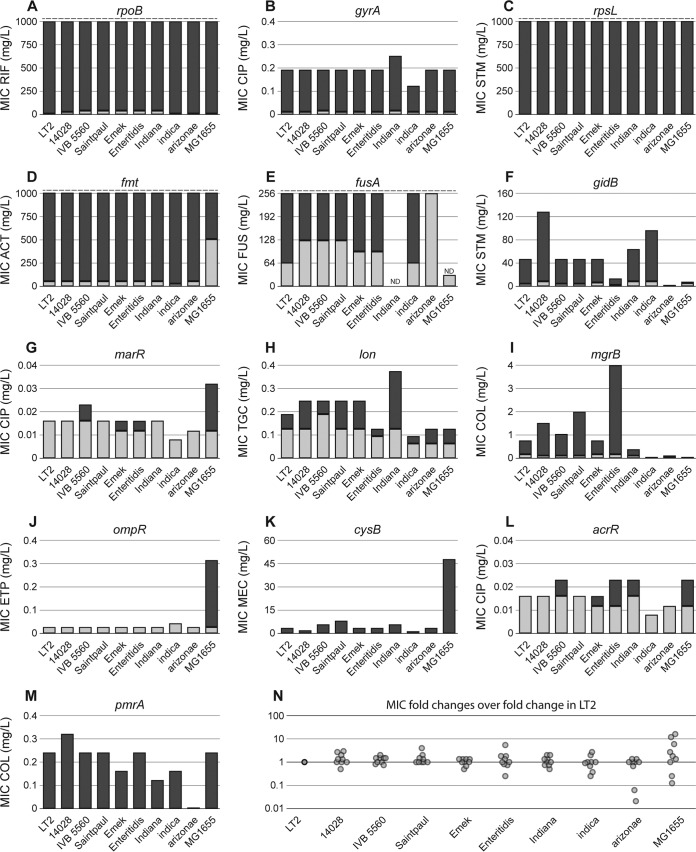
Effects of mutant alleles on antibiotic resistance in different genetic backgrounds. (A to M) MICs of the different antibiotics for each strain carrying the indicated mutant allele and the respective wild-type strain. Light gray bars indicate the MIC of the wild-type strain, and dark gray bars represent the MIC of the mutant. Dashed lines indicate the detection limit. Two MICs for *fusA* P413L could not be determined due to strain construction difficulties (*S*. Indiana) or lethality on plates containing EDTA (E. coli *fusA* P413L). (N) Fold change difference of mutations introduced in the indicated strain over the fold change difference that the mutation causes in *S*. Typhimurium LT2. For complete strain designations and phylogenetic relationships, see Fig. 3 and Table S1. Abbreviations: RIF, rifampin; CIP, ciprofloxacin; STM, streptomycin; ACT, actinonin; FUS, fusidic acid; TGC, tigecycline; COL, colistin; ETP, ertapenem; MEC, amdinocillin; ND, not determined.

To investigate the influence of the genetic context on the phenotypic expression of chromosomal resistance mutations, we determined the MIC values and exponential growth rates of the 13 resistance alleles when present in 10 different strain backgrounds (except for *fusA* P413L which was constructed in only nine strains) (Fig. 4 and 5). The effects of target alteration mutations (in the *rpsL*, *gyrA*, *rpoB*, *fmt*, *fusA*, and *gidB* genes) on exponential growth rate were very consistent among all backgrounds. Thus, mutants that were previously described as cost free (36, 37) did not exhibit growth defects in any of the 10 investigated genetic backgrounds. The biggest variation was seen for the target mutation with the strongest impact on fitness, *fmt* T12R (Fig. 4).

Similar to the strain independence of fitness effects, we observed very little variation in the effects on antibiotic resistance levels by target alteration mutations in the different host bacteria. Mutations in *rpoB*, *rpsL*, and *fmt* caused high-level resistance to rifampin, streptomycin, and actinonin exceeding 1 g/liter, which is the detection limit of Etests. Since these concentrations are close to the antibiotic solubility limits, we could not test higher ranges using microdilution assays and were therefore unable to determine the MIC. Similar results were obtained for *fusA* P413L, which caused resistance exceeding the detection limit of the Etest strip. We also could not determine the MIC of fusidic acid in S. enterica subsp. arizonae, because the parental strain with a wild-type *fusA* allele was already fully resistant. The resistance increase to fluoroquinolone provided by *gyrA* S83F was almost identical in all host strains, and only the *gidB* Q167* mutant showed strong differences in resistance levels (Fig. 5).

### Strain-specific effects of regulator mutations.

Among the seven regulator mutations included in this study, five (*acrR*, *marR*, *mgrB*, *pmrA*, and *ompR*) did not show a growth defect in *S*. Typhimurium LT2 or in any of the other host strains with the exception of deletion of *ompR* which was strongly deleterious in E. coli MG1655 but did not affect the other host strains. Besides *ompR*, a deletion of *cysB* had a much bigger deleterious effect in E. coli MG1655 compared to other hosts. The most diverse effect was exhibited by *lon* Q137*. In three strains, it imposed only a very small fitness cost, whereas in other strains, the fitness cost was higher. In addition, all *lon* mutants exhibited mucoidy, a known phenotype for *lon* deficient strains (33). The strongest phenotypic variation in this study was observed for the effects of regulator mutations on antibiotic resistance. Thus, mutations in *marR*, *lon*, *mgrB*, *ompR*, *cysB*, and *pmrA* showed up to >500-fold differences in resistance level, depending on the strain background (Fig. 5).

## DISCUSSION

### Combinations of resistance mutations cause predictable phenotypes.

Understanding potential epistatic interactions between different resistance mutations is important for our ability to forecast the dynamics of resistance development. Here we show that the fitness effects of combinations of up to four mutations had largely predictable additive effects. Thus, for 24/29 mutants, the measured exponential growth rates were close to the additive values of the individual mutants. Only 3/29 mutants showed growth rates higher than additive values, and only 2 of these mutants showed sign epistasis. This low frequency of positive epistatic interactions (10%) is lower than in other studies (24, 27, 29, 38). For our two cases as well as for the previously reported examples, the underlying mechanistic basis of the sign epistasis is not well understood and requires further study.

The relative rarity of positive epistasis in our study compared to previous work can be attributed to several possible factors. (i) The mentioned studies report a strong allele specificity of epistatic interactions. While, for example, combinations of some mutant *gyrA* and *rpoB* alleles revealed sign epistasis, others behaved additively. It is possible, albeit unlikely, that the alleles used in our study happen to be unaffected by epistatic interactions. The fact that we do see epistatic interactions in specific cases involving mutant alleles of *rpoB*, *rpsL*, *fusA*, *fmt*, *ompR*, and *lon* suggests that a general absence of epistasis is unlikely to be the explanation for our observations. (ii) Unlike other studies, we used *S*. Typhimurium LT2 as a host organism for studying epistatic interactions. The genetic background might play a central role in inhibiting or promoting epistatic interactions between different alleles. (iii) Most studies rely on selection coefficients obtained from competition experiments as a measure of fitness, but our fitness measurements are exponential growth rates, which might also account for some of the differences from previous studies. (iv) Finally, to our knowledge, this is the first study on epistatic interactions between resistance mutations where the whole genome of each constructed strain was sequenced to ensure the absence of second-site mutations. Strain construction by lambda red recombineering or transduction involve multiple growth cycles in liquid and on plates easily exceeding 50 generations of growth until a fitness assay can be conducted. These periods of growth can rapidly select for compensatory mutations that increase growth rates, especially in the case of mutations that severely impact fitness. If this growth compensation by off-target mutations occurs before the fitness measurement is performed, the increased fitness will be falsely attributed to positive epistatic interactions between the investigated resistance mutations. In fact, we had to reconstruct several strains because we detected off-target mutations after whole-genome sequencing, indicating that this is a relatively common problem and that it might cause a significant overestimation of positive epistasis. One method used by some researchers is to make controls with several parallel constructions of the same strains, and if similar results are obtained for all parallel constructs, it is assumed that no compensation has occurred. However, this approach is not fool proof, since if the fitness of the constructed strains is low and off-target compensatory mutations are common, they could repeatedly occur also in parallel constructs. Thus, the whole genomes of strains used for fitness assays ought to be sequenced to avoid off-target compensation during construction.

Our combinatorial sets of mutations were focused on epistatic interactions either between target alteration mutation or regulator mutations selected on different antibiotics with one mutant allele per gene. This constraint was necessary to be able to sequence the whole genome of every constructed strain. Future studies on epistatic interactions between (i) target and regulator mutations, (ii) multiple alleles per gene, and (iii) epistatic interactions between mutations selected on the same antibiotic are necessary to further elucidate the epistatic robustness of chromosomal resistance mutations. Decreasing future costs of whole-genome sequencing will provide the ability for broader screens, including the factors mentioned above.

### The phenotypic effects of resistance mutations are largely strain independent.

One novel aspect of epistatic interactions that was addressed here is how does the genetic background of an organism affect the phenotypic expression of a resistance mutation? Considering that the presence or absence of an individual mutation can determine whether a second mutation is deleterious or beneficial, the potential for epistasis of single mutations that occur in two different strains with thousands of genetic changes ought to be high. In fact, a recent study in *Pseudomonas* shows that for 50% of *rpoB* mutations, the fitness cost is dependent on the strain background (31). The observed phenotype could be attributed to different transcriptional efficiencies in the host strains. Given that central cellular processes like transcription rates are heterogeneous in closely related strains, it is possible that strain dependence is common among chromosomal resistance mutations. However, the broad analysis performed here of 13 mutations associated with transcription, translation, replication, and drug influx and efflux in 10 different strain backgrounds shows a different picture. Thus, all target alteration mutations had very robust phenotypes and were largely unaffected by the genetic background. The impact on growth and resistance was highly predictable, with the exception of *gidB* Q167*, which did not increase resistance in *S. enterica* subsp. *arizonae* and E. coli MG1655. It was shown previously that the increase of resistance due to the loss of GidB is largely dependent on the presence of the aminoglycoside adenyltransferase AadA in *S*. Typhimurium LT2 (39). Notably, both *S. enterica* subsp. *arizonae* and E. coli lack this enzyme, while all other strains included in this study encode a functional *aadA* gene, which might explain the difference in the phenotypic expression of GidB loss.

The deleterious effects imposed by regulator mutations were also mostly similar across the strains but also had some exceptions. For example, a deletion of *ompR*, which encodes a transcription factor associated with regulation of outer membrane proteins (40), was strongly deleterious in E. coli MG1655 but neutral in all tested *Salmonella* strains. We previously showed that the cost associated with porin loss is strictly dependent on the presence or absence of alternative porins (12). Therefore, one explanation for the strong strain dependence of *ompR* deletions could be differences in the outer membrane profiles of the host strains, where strains with a higher expression of alternative porins buffer the effect of OmpR loss. This correlated with the observation that OmpR loss caused an increased resistance only in E. coli and not in any other strain. Additionally, it is possible that expression of OmpC and OmpF in *Salmonella* is less dependent on the presence of OmpR, since the OmpR orthologues of E. coli and *Salmonella* functionally diverged (41). While most mutations showed a consistent effect on resistance across the majority of host strains, we observed some outliers of this trend in almost all sets of mutant regulator alleles (Fig. 5).

In summary, we show that effects on exponential growth rates and antibiotic resistance levels are highly predictable (i) for combinations of different resistance mutations and (ii) across several different host strains, indicating that in most cases, we can predict the fitness effects of a single resistance mutation in combination with other resistance mutations and extrapolate results from one specific strain to different genetic backgrounds. However, this generalization was not without exceptions, since a small fraction of specific host/mutation combinations revealed deviations in both resistance and fitness, highlighting the importance of the choice of the host bacterium when studying resistance mutations. Although we did not observe a correlation between phenotypic diversity with regard to fitness or resistance level among the tested *Salmonella* strains, the diversity increased considerably when we included E. coli MG1655 as a representative of a different species (Fig. 4 and 5). While we cannot conclude that this effect is based on genetic distance, it shows that studies of epistatic interactions of antibiotic resistance mutations with a more diverse set of host strains, including multiple genera is required to elucidate limitations to the observed epistatic robustness.

Finally, even though the specific resistance mutations studied here are of limited clinical relevance in Salmonella (except for *gyrA* mutations and fluoroquinolone resistance), the data we have on epistatic interactions are important in that they show how the phenotypes in a model system are largely predictable. This implies that these findings might be applicable to other pathogens (e.g., Mycobacterium tuberculosis) where clinical resistance is caused by different chromosomal mutations rather than by plasmid-borne genes. Such predictability of the effect of combinations of chromosomal mutations on fitness/resistance will improve our ability to forecast the emergence and spread of multidrug-resistant bacteria and to identify which drug combinations are most efficient in reducing resistance evolution.

## MATERIALS AND METHODS

### Strains and growth conditions.

The strains used in this study are listed in Table S3 in the supplemental material. Unless indicated otherwise, bacteria were grown in lysogeny broth (LB) containing 10 g/liter NaCl, 10 g/liter tryptone, and 5 g/liter yeast extract at 37°C and 200 rpm shaking. For growth on plates, LB was supplemented with 15 g/liter agar (LA). If needed, the growth medium was supplemented with 15 mg/liter chloramphenicol, 50 mg/liter kanamycin, or 15 mg/liter tetracycline for selection of genetic markers or 50 g/liter sucrose for counterselection of the *sacB* gene.

10.1128/mBio.00770-18.8 TABLE S3  Strains used in this study. Download TABLE S3, DOCX file, 0.1 MB. Copyright © 2018 Knopp and Andersson. 2018 Knopp and Andersson This content is distributed under the terms of the Creative Commons Attribution 4.0 International license.

10.1128/mBio.00770-18.9 TABLE S4  Average nucleotide identities (ANIs). White boxes show the ANIs of all strains used in this study. Gray boxes show the corresponding standard deviations. Download TABLE S4, DOCX file, 0.1 MB. Copyright © 2018 Knopp and Andersson. 2018 Knopp and Andersson This content is distributed under the terms of the Creative Commons Attribution 4.0 International license.

### Calculation of average nucleotide identities.

Average nucleotide identities (ANI) were calculated as described by Goris et al. (42) using the ANI calculator provided by Kosta Konstantinidis (http://enve-omics.ce.gatech.edu/ani/). The minimum length of the alignment was set at 700 nucleotides (nt) with a minimum identity of 70%. The genomes were fragmented using a window size of 1,000 bp and a step size of 200 bp. Whole-genome sequences were obtained either online (E. coli MG1655 [GenBank accession no. U00096] and *S*. Typhimurium LT2 [GenBank accession no. AE006468]) or generated in this study.

### Construction of the phylogenetic tree.

The concatenated sequences of the seven housekeeping genes *hisD*, *purE*, *sucA*, *thrA*, *aroC*, *dnaN*, and *hemD* were aligned using MAFFT v7.305b (43) with the L-INS-i algorithm. The maximum likelihood tree was reconstructed based on the alignment with IQ-TREE v1.6.1 (44), using ModelFinder (45) to identify the most appropriate model. One thousand bootstraps were drawn with the ultrafast bootstrap approximation (46). The model with the best fit was TIM+F+R3 (transition model [AC=GT and AT=CG]) with unequal base frequencies, using empirical base frequencies and the FreeRate model of heterogeneity across sites, with three categories). The phylogenetic tree was visualized using FigTree 1.4.3 (http://tree.bio.ed.ac.uk/software/figtree) and Adobe Illustrator.

### Strain construction.

All mutant alleles were constructed in *S*. Typhimurium LT2, *S*. Saintpaul, and E. coli MG1655 with λ red recombineering using pSIM5-tet (tet stands for tetracycline) (47, 48). For this, bacteria were grown overnight at 30°C with 15 mg/liter tetracycline, diluted 1:100 in LB supplemented with 15 mg/liter tetracycline, and grown at 30°C with constant shaking to an optical density at 600 nm (OD_600_) of 0.2. The cultures were moved to 42°C to induce the temperature-controlled λ red genes. After 15 min, the cultures were quickly cooled down on ice and washed three times with 10% glycerol. Pellets were resuspended in glycerol, and aliquots were mixed with DNA in a chilled Eppendorf tube. Prior to electroporation 50 µl of the cell/DNA mix was transferred to an electroporation cuvette (1-mm gap), and electroporation was performed with a Gene Pulser (Bio-Rad) at 1.9 kV, 400 Ω, and 25 µF. The cells were quickly transferred into 1 ml prewarmed LB and recovered for at least 1 h at 30°C. Counterselection was typically recovered overnight to allow segregation and degradation of SacB.

In general, two strategies were used to construct mutants. In the case of *rpsL*, *rpoB*, *gyrA*, *fusA*, and *fmt*, single-stranded DNA oligonucleotides containing the mutation and 35-nucleotide (nt) upstream and downstream homologous sequences were used to transform electrocompetent cells. After recovery, the cells were plated on LA containing 200 mg/liter streptomycin (*rpsL* K42N), 100 mg/liter rifampin (RIF) (*rpoB* S531L), 100 mg/liter fusidic acid and 1 mM EDTA (*fusA* P413L), 0.1 mg/liter ciprofloxacin (*gyrA* S83F), or 200 mg/liter actinonin (*fmt* T12R). In the case of E. coli MG1655. successful transformants carrying the *fmt* T12R allele were screened without selection, since the wild type also showed growth on selective plates. All other mutations were constructed by introduction of a *cat*-*sacB* cassette into the gene of interest by amplifying the cassette with 35-nt overhangs homologous to the target region. Transformants were selected on LA supplemented with 15 mg/liter chloramphenicol. In a second round of λ red recombineering using a single-stranded DNA oligonucleotide containing the target mutation, the *cat*-*sacB* cassette was removed by counterselection on LA supplemented with 50 g/liter sucrose. All strains were verified by PCR and subsequent sequencing.

After all mutant alleles were constructed, we used the duplication-insertion recombineering method to force a duplication of the mutant allele as previously described (49). In short, a *cat*-*sacB* cassette was used to introduce a tandem duplication, which was subsequently transduced into the target *Salmonella* strains using P22 HT *int* phage transduction (phage P22 mutant with increased transduction abilities). The transductants were subsequently grown on LA supplemented with 50 g/liter sucrose to segregate the duplication, leaving the mutation of interest inserted in the target gene. The sequences of all mutants were verified. In the case of *S. enterica* subsp. *arizonae* and *S. enterica* subsp. *indica*, we first introduced the cosmid pPR1347 into the host strains by transformation and selection on LA supplemented with 50 mg/liter kanamycin (34). As long as selection was maintained, the cells expressed the O antigen of *S*. Typhimurium LT2, causing P22 susceptibility. After transduction, the cosmid was rapidly lost by removal of selection. The complete cloning strategy is illustrated in Fig. S4 and S5.

### Sequencing.

Genomic DNA was extracted using the MasterPure DNA purification kit (Epicentre, USA) according to the manufacturer’s recommendations. The samples were subjected to whole-genome sequencing using a MiSeq system (Illumina, USA). The paired-end sequence reads were trimmed and mapped using CLC Genomics Workbench (CLC Bio, Denmark) using standard parameters. Subsequently, inversion, deletions, structural variants, and single nucleotide polymorphisms were determined using standard parameters. Paired-end sequence reads of the host strains are accessible at the NCBI sequence read archive (SRA) under BioProject identifier (ID) or accession no. PRJNA450591.

### Determination of MIC.

MICs were determined using Etest strips (bioMérieux, France) on Mueller-Hinton agar plates as recommended by the manufacturer. For slow-growing mutants, the incubation period was prolonged until the inhibition zones were clearly visible. For fusidic acid Etests, the plates were supplemented with 5 mM EDTA to increase sensitivity. Colistin MICs were additionally determined on Mueller-Hinton II (cation-adjusted, Becton Dickinson) agar with a lowered pH of 5.5. MICs for actinonin and rifampin were determined using microdilutions of the corresponding antibiotic. Approximately 10^5^ cells were inoculated with twofold dilutions of actinonin or rifampin in a 96-well plate at a final volume of 200 µl and incubated for approximately 20 h or until growth was visible. The concentration with no visible growth was determined to be the MIC.

### Determination of maximum exponential growth rate.

Growth rates were determined using a Bioscreen C reader (Oy Growth Curves Ab Ltd., Finland). A minimum of four biological replicates were grown overnight and diluted 1:1,000 in LB medium, and two 300-µl aliquots of each replicate were transferred to honeycomb plates. The plates were incubated in the Bioscreen C reader for 18 h at 37°C and continuous shaking. OD_600_ values were measured in 4-min intervals. The exponential growth rate was calculated based on the OD_600_ interval 0.024 to 0.09 and normalized to the growth rate of the corresponding wild-type strain, which was included on each plate.

### Calculation of epistasis.

Pairwise epistasis (ε) was determined as previously described (24, 30) assuming a multiplicative model of allelic interaction, in which ε_*AB*_ = *W_AB_W_ab_* − *W_Ab_W_aB_*, where *W*_*ij*_ is the fitness of strain carrying two mutant alleles and *W*_*IJ*_ denotes a strain with both wild-type alleles. In the case of ε_*AB*_ = 0, no espistatic interactions can be observed between the two alleles, while ε_*AB*_ > 0 and ε_*AB*_ < 0 indicate positive and negative epistatic interactions, respectively. Sign epistasis is observed when *W*_*AB*_ is higher than *W*_*A*_ and/or *W*_*B*_. The theoretical fitness of a double mutant assuming no epistasis (ε_*AB*_ = 0) was determined by multiplying the fitness of both single mutants (*W*_*AB*_expected_ = *W_Ab_W_aB_*). To determine whether the actual value significantly deviates from the expected value, we performed a *t* test where the standard deviation of the theoretical value representing no epistatic interactions was propagated from the standard deviation (δ) of the single mutants using the formula:

$$\frac{W_{AB\_\text{exp}}}{W_{AB\_\text{exp}}}=\sqrt{{\left(\frac{\delta W_{A}}{W_{A}}\right)}^{2}+{\left(\frac{\delta W_{B}}{W_{B}}\right)}^{2}}$$

where *W*_*AB*___exp_ is the expected fitness of a strain with two wild-type alleles. The expected fitness assuming no epistatic interactions between more than two alleles was calculated as previously described (50). Growth rates, including standard deviations, epistatic terms, and projected fitness of the combinatorial mutants, are listed in Table S5.

10.1128/mBio.00770-18.10 TABLE S5  Relative growth values, standard deviations, epistatic values, and nonepistatic estimates for the combinatorial mutant sets. Download TABLE S5, DOCX file, 0.1 MB. Copyright © 2018 Knopp and Andersson. 2018 Knopp and Andersson This content is distributed under the terms of the Creative Commons Attribution 4.0 International license.
