# Supplementary figures and images for: Predictable Phenotypes of Antibiotic Resistance Mutations
Source: mBio. 2018 May 15;9(3):e00770-18. doi: 10.1128/mBio.00770-18 (PMC5954217; doi:10.1128/mBio.00770-18)

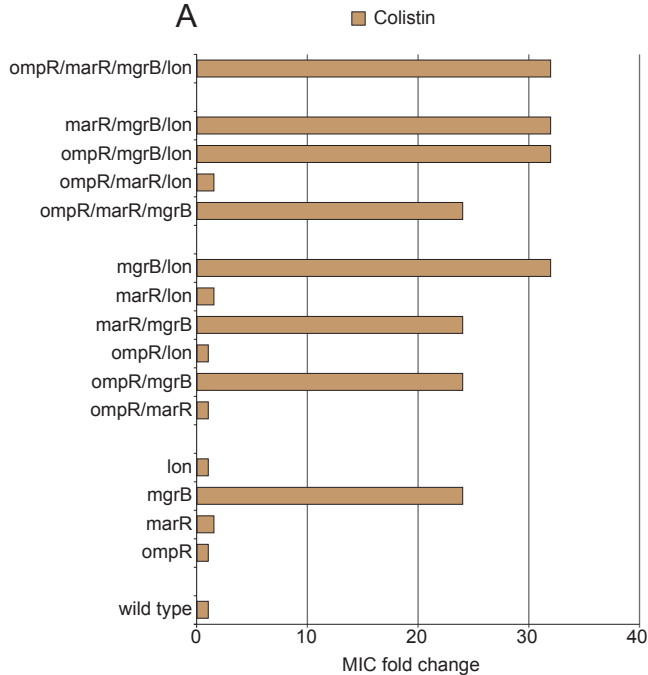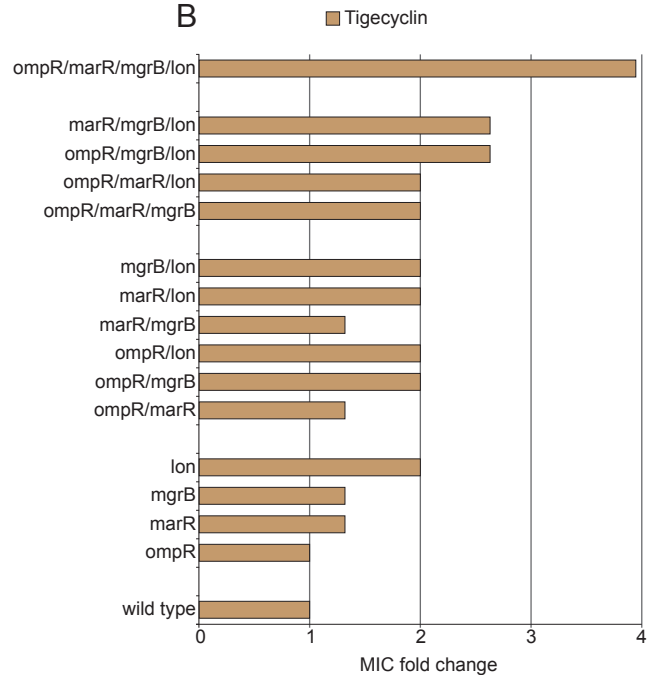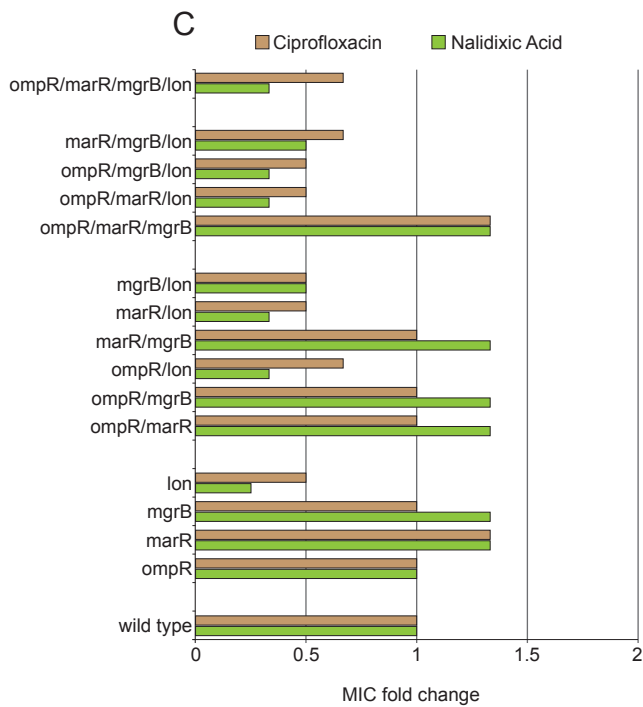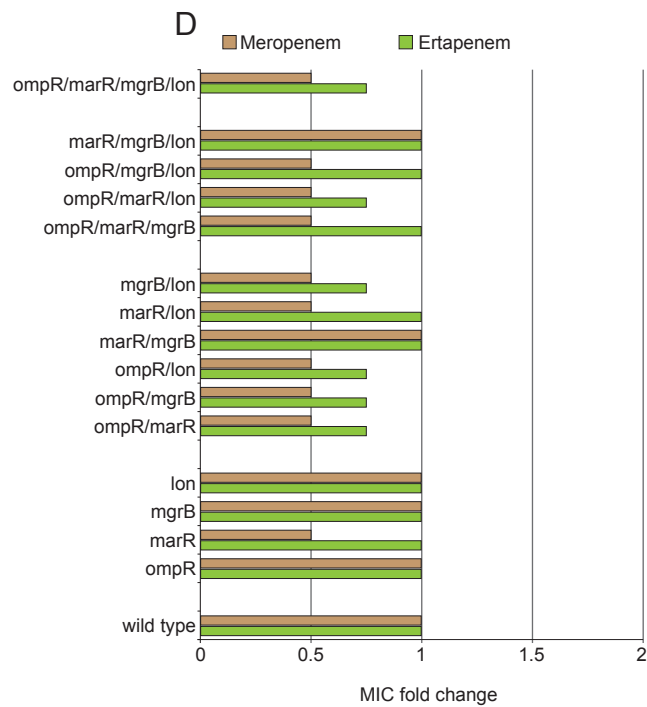

Supplement: FIG S1 [file mbo003183881sf1.pdf]

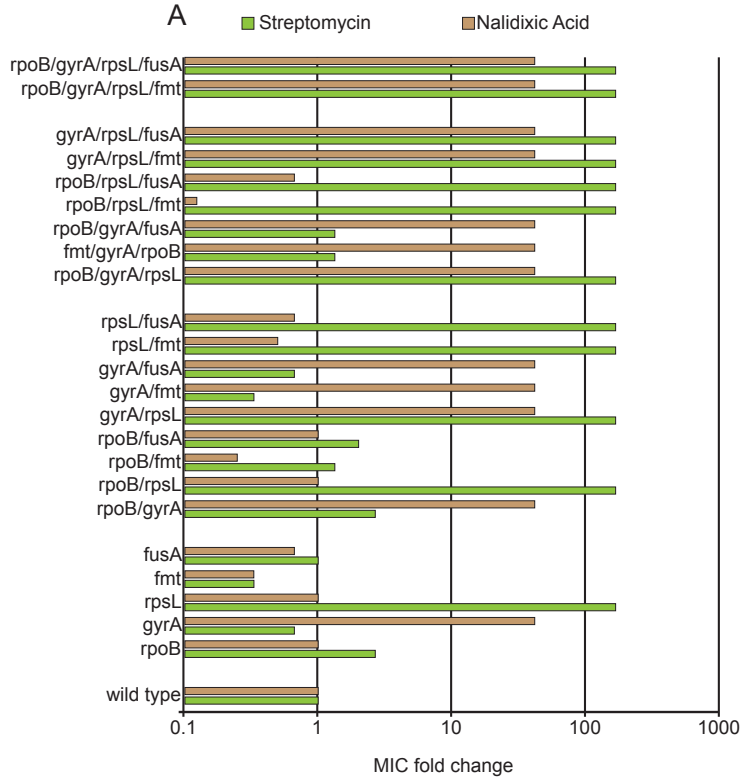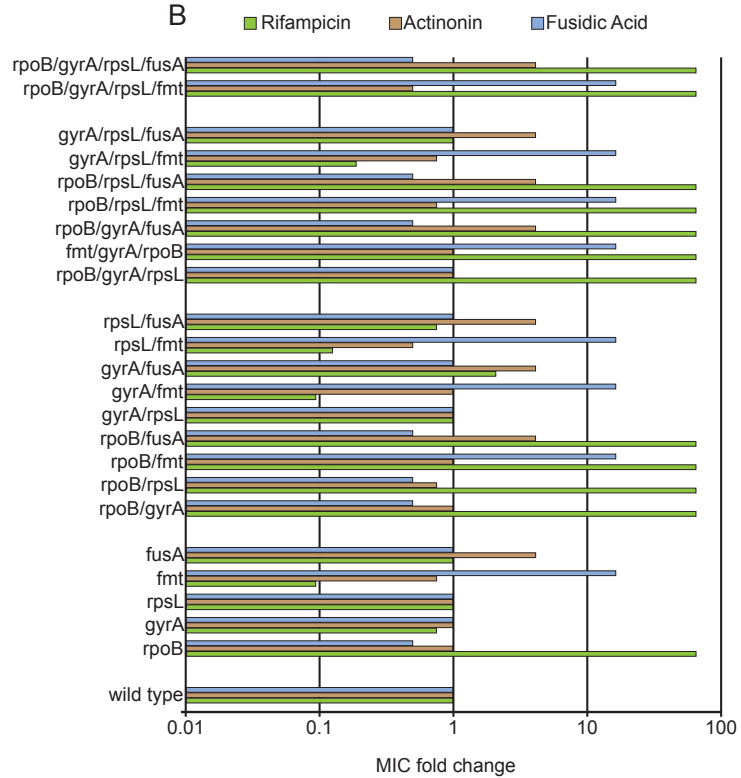

Supplement: FIG S2 [file mbo003183881sf2.pdf]

relative exponential growth rate

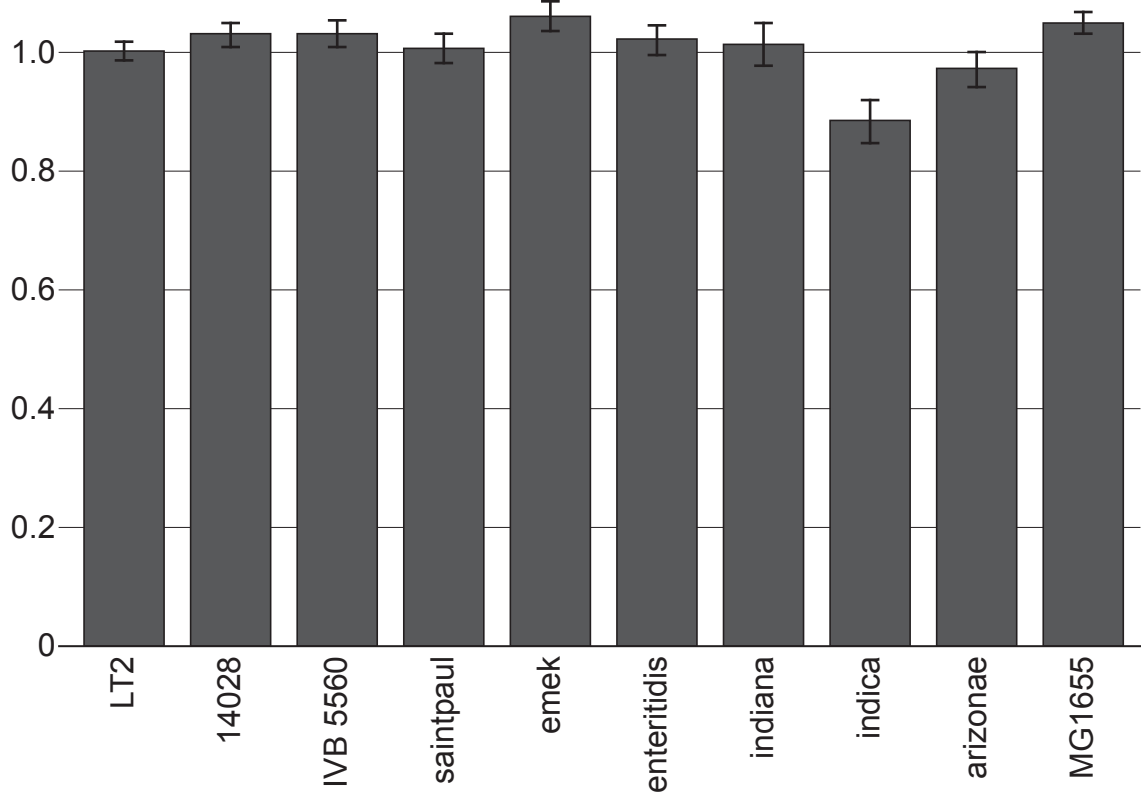

Supplement: FIG S3 [file mbo003183881sf3.pdf]

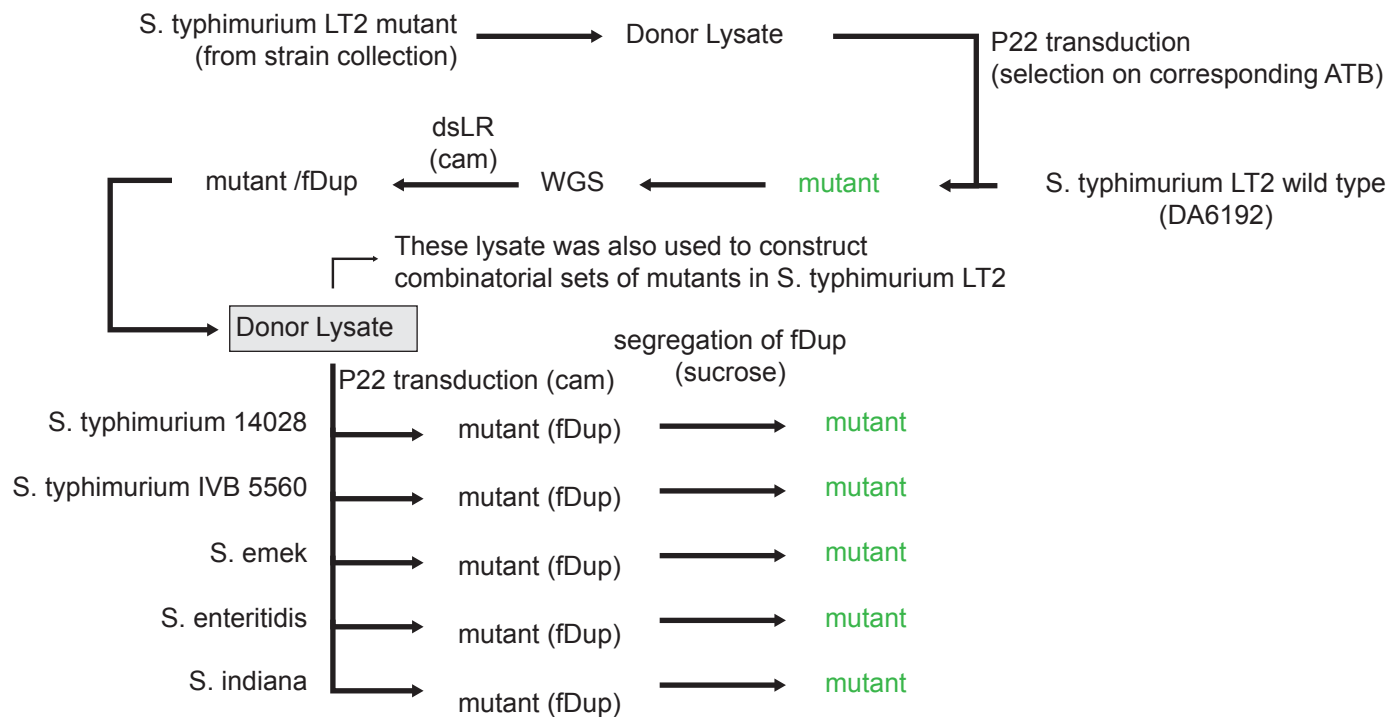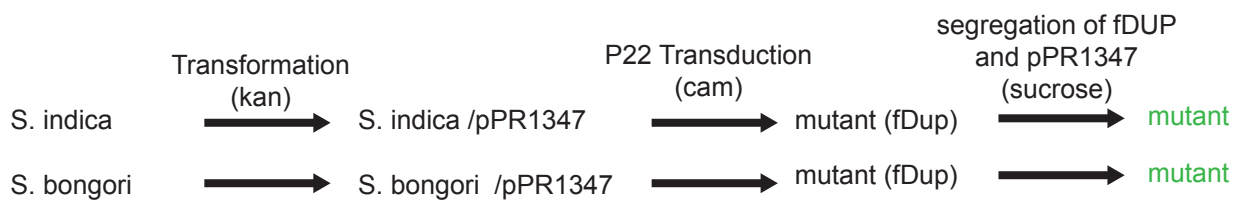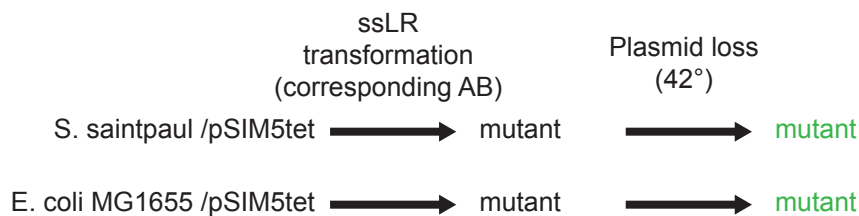

Supplement: FIG S4 [file mbo003183881sf4.pdf]

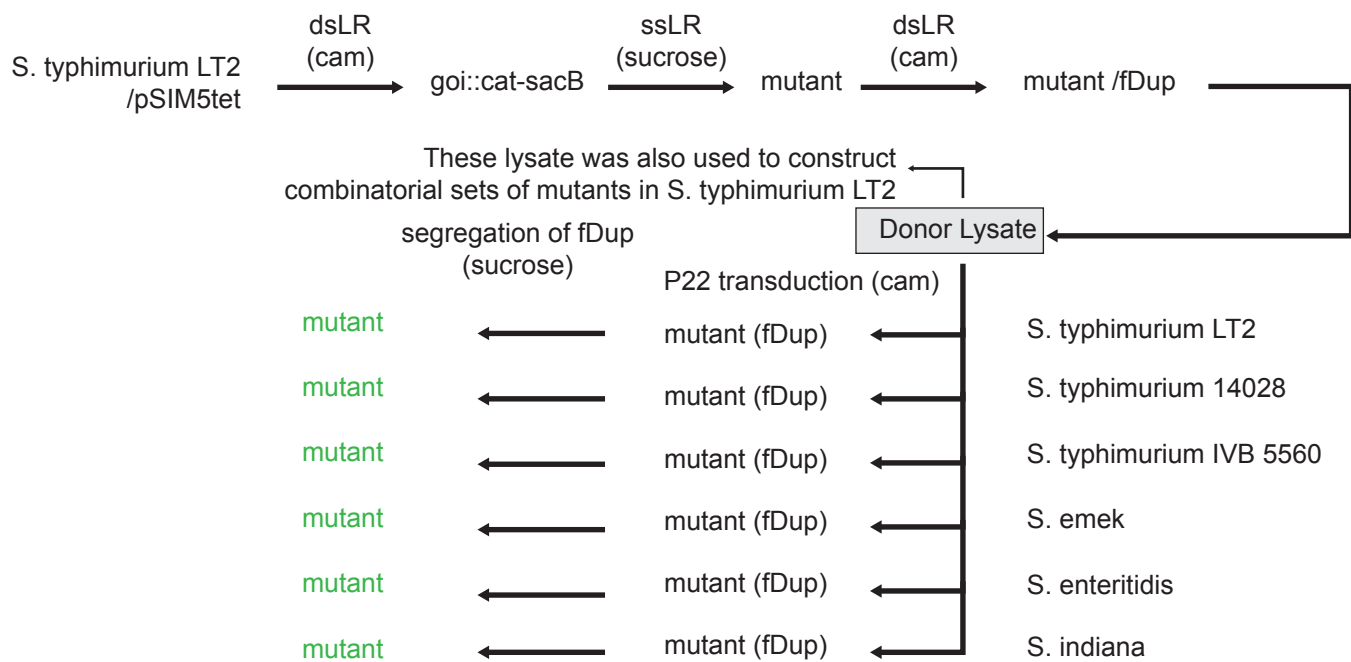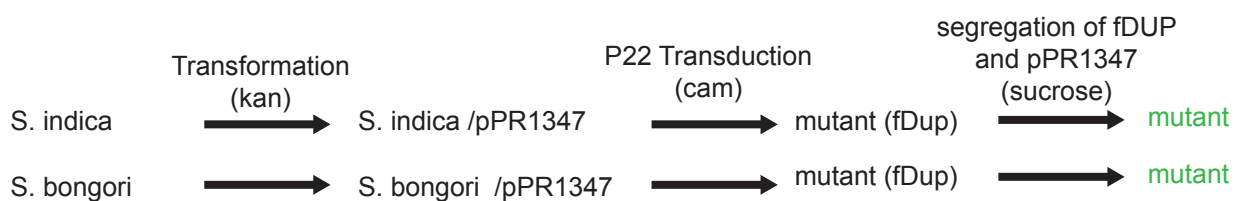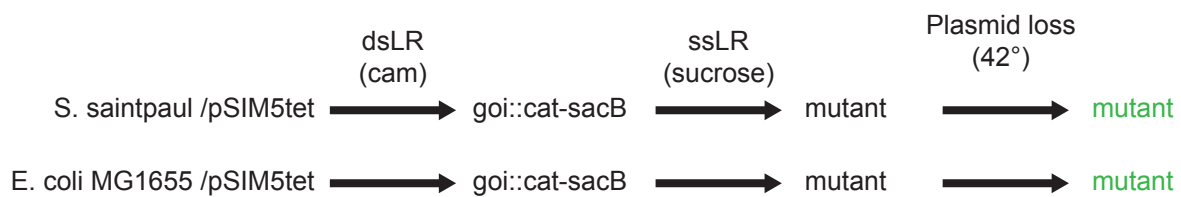

Supplement: FIG S5 [file mbo003183881sf5.pdf]
