# Supplementary material for: Predictable Phenotypes of Antibiotic Resistance Mutations
Source: mBio. 2018 May 15;9(3):e00770-18. doi: 10.1128/mBio.00770-18 (PMC5954217; doi:10.1128/mBio.00770-18)
Supplement: TABLE S1 [file mbo003183881st1.docx]

| **Abbreviation** | **Complete designation** |
| --- | --- |
| *S. typhimurium* LT2 | *Salmonella enterica* subsp. *enterica* serovar Typhimurium strain LT2 |
| *S. typhimurium* 14028 | *Salmonella enterica* subsp. *enterica* serovar Typhimurium strain 14028 |
| *S. typhimurium* IVB 5560 | *Salmonella enterica* subsp. *enterica* serovar Typhimurium strain IVB 5560 |
| *S.* Saintpaul | *Salmonella enterica* subsp. *enterica* serovar Saintpaul CDC B1605 |
| *S.* Emek | *Salmonella enterica* subsp. *enterica* serovar Emek IVB4793/3366 |
| *S.* Enteritidis | *Salmonella enterica* subsp. *enterica* serovar Enteritidis IVB 470/82 |
| *S.* Indiana | *Salmonella enterica* subsp. *enterica* serovar Indiana DMS 3702 |
| *S. indica* | *Salmonella enterica* subsp. *indica* ATCC 43976 |
| *S. arizonae* | *Salmonella enterica* subsp. *arizonae* ATCC 13314 |
| *E. coli* MG1655 | *Escherichia coli* K12 MG1655 |
