# Supplementary material for: Predictable Phenotypes of Antibiotic Resistance Mutations
Source: mBio. 2018 May 15;9(3):e00770-18. doi: 10.1128/mBio.00770-18 (PMC5954217; doi:10.1128/mBio.00770-18)
Supplement: TABLE S2 [file mbo003183881st2.docx]

| **Host** | **Transferred**  **mutation** | **Fragment**  **Size (base pairs)** | **Single Nucleotide Polymorphisms** | |
| --- | --- | --- | --- | --- |
|  |  |  | **synonymous** | **non synonymous** |
| *S.* Enteritidis | *marR* H110* | 4596 - 3860 | 26 | 2 |
| *S.* Emek | *marR* H110* | 5606 - 4927 | 30 | 3 |
| *S.* *indica* | *marR* H110* | 16307 - 16119 | 483 | 79 |
| *S.* *arizonae* | *marR* H110* | 5708 - 5592 | 164 | 52 |
| *S.* Enteritidis | ∆*ompR* | 1819 - 296 | 4 | 0 |
| *S.* Emek | ∆*ompR* | 25039 - 24908 | 142 | 38 |
| *S.* *indica* | ∆*ompR* | 10120 - 9900 | 254 | 63 |
| *S.* *arizonae* | ∆*ompR* | 11492 - 11202 | 473 | 38 |
