# Supplementary material for: Predictable Phenotypes of Antibiotic Resistance Mutations
Source: mBio. 2018 May 15;9(3):e00770-18. doi: 10.1128/mBio.00770-18 (PMC5954217; doi:10.1128/mBio.00770-18)
Supplement: TABLE S3 [file mbo003183881st3.docx]

| **Strain Number** | **Organism** | **Genotype** | **Source** |
| --- | --- | --- | --- |
| DA6192 | *S. typhimurium* LT2 | wild type | Strain collection |
| DA27238 | *S. typhimurium* LT2 | /pSIM5-tet | Strain collection |
| DA46691 | *S. typhimurium* LT2 | *gyrA* S83F | This study |
| DA28104 | *S. typhimurium* LT2 | *rpoB* S531L | This study |
| DA26816 | *S. typhimurium* LT2 | *rpsL* K42N | This study |
| DA46690 | *S. typhimurium* LT2 | *fusA* P413L | This study |
| DA46692 | *S. typhimurium* LT2 | *fmt* T12R | This study |
| DA48709 | *S. typhimurium* LT2 | *gidB* Q167* | This study |
| DA48707 | *S. typhimurium* LT2 | ∆*mgrB* | This study |
| DA48715 | *S. typhimurium* LT2 | *acrR* H115Y | This study |
| DA48713 | *S. typhimurium* LT2 | *marR* Q110* | This study |
| DA48717 | *S. typhimurium* LT2 | ∆*ompR* | This study |
| DA48711 | *S. typhimurium* LT2 | *lon* Q137* | This study |
| DA48719 | *S. typhimurium* LT2 | ∆*cysB* | This study |
| DA51679 | *S. typhimurium* LT2 | *pmrA* G53E | This study |
| DA42200 | *S. typhimurium* 14028 | wild type | Strain collection |
| DA28118 | *S. typhimurium* 14028 | *gyrA* S83F | This study |
| DA28106 | *S. typhimurium* 14028 | *rpoB* S531L | This study |
| DA26819 | *S. typhimurium* 14028 | *rpsL* K42N | This study |
| DA28132 | *S. typhimurium* 14028 | *fusA* P413L | This study |
| DA32280 | *S. typhimurium* 14028 | *fmt* T12R | This study |
| DA48750 | *S. typhimurium* 14028 | *gidB* Q167* | This study |
| DA48748 | *S. typhimurium* 14028 | ∆*mgrB* | This study |
| DA48756 | *S. typhimurium* 14028 | *acrR* H115Y | This study |
| DA48754 | *S. typhimurium* 14028 | *marR* Q110* | This study |
| DA48758 | *S. typhimurium* 14028 | ∆*ompR* | This study |
| DA48752 | *S. typhimurium* 14028 | *lon* Q137* | This study |
| DA48760 | *S. typhimurium* 14028 | ∆*cysB* | This study |
| DA51681 | *S. typhimurium* 14028 | *pmrA* G53E | This study |
| DA9865 | *S. typhimurium* IVB 5560 | wild type | Strain collection |
| DA28120 | *S. typhimurium* IVB 5560 | *gyrA* S83F | This study |
| DA28110 | *S. typhimurium* IVB 5560 | *rpoB* S531L | This study |
| DA26817 | *S. typhimurium* IVB 5560 | *rpsL* K42N | This study |
| DA28135 | *S. typhimurium* IVB 5560 | *fusA* P413L | This study |
| DA32282 | *S. typhimurium* IVB 5560 | *fmt* T12R | This study |
| DA48764 | *S. typhimurium* IVB 5560 | *gidB* Q167* | This study |
| DA48762 | *S. typhimurium* IVB 5560 | ∆*mgrB* | This study |
| DA48770 | *S. typhimurium* IVB 5560 | *acrR* H115Y | This study |
| DA48768 | *S. typhimurium* IVB 5560 | *marR* Q110* | This study |
| DA48772 | *S. typhimurium* IVB 5560 | ∆*ompR* | This study |
| DA48766 | *S. typhimurium* IVB 5560 | *lon* Q137* | This study |
| DA48774 | *S. typhimurium* IVB 5560 | ∆*cysB* | This study |
| DA51684 | *S. typhimurium* IVB 5560 | *pmrA* G53E | This study |
| DA9879 | *S.* Saintpaul | wild type | Strain collection |
| DA21666 | *S.* Saintpaul | /pSIM5-tet | Strain collection |
| DA28122 | *S.* Saintpaul | *gyrA* S83F | This study |
| DA28109 | *S.* Saintpaul | *rpoB* S531L | This study |
| DA28156 | *S.* Saintpaul | *rpsL* K42N | This study |
| DA28136 | *S.* Saintpaul | *fusA* P413L | This study |
| DA32284 | *S.* Saintpaul | *fmt* T12R | This study |
| DA47566 | *S.* Saintpaul | *gidB* Q167* | This study |
| DA46338 | *S.* Saintpaul | ∆*mgrB* | This study |
| DA47180 | *S.* Saintpaul | *acrR* H115Y | This study |
| DA47303 | *S.* Saintpaul | *marR* Q110* | This study |
| DA47212 | *S.* Saintpaul | ∆*ompR* | This study |
| DA48114 | *S.* Saintpaul | *lon* Q137* | This study |
| DA48120 | *S.* Saintpaul | ∆*cysB* | This study |
| DA49603 | *S.* Saintpaul | *pmrA* G53E | This study |
| DA9945 | *S.* Enteritidis | wild type | Strain collection |
| DA49894 | *S.* Enteritidis | *gyrA* S83F | This study |
| DA49896 | *S.* Enteritidis | *rpoB* S531L | This study |
| DA49898 | *S.* Enteritidis | *rpsL* K42N | This study |
| DA49900 | *S.* Enteritidis | *fusA* P413L | This study |
| DA49902 | *S.* Enteritidis | *fmt* T12R | This study |
| DA51843 | *S.* Enteritidis | *gidB* Q167* | This study |
| DA49184 | *S.* Enteritidis | ∆*mgrB* | This study |
| DA49148 | *S.* Enteritidis | *acrR* H115Y | This study |
| DA49152 | *S.* Enteritidis | *marR* Q110* | This study |
| DA49150 | *S.* Enteritidis | ∆*ompR* | This study |
| DA49186 | *S.* Enteritidis | *lon* Q137* | This study |
| DA50184 | *S.* Enteritidis | ∆*cysB* | This study |
| DA51685 | *S.* Enteritidis | *pmrA* G53E | This study |
| DA9949 | *S.* Emek | wild type | Strain collection |
| DA49904 | *S.* Emek | *gyrA* S83F | This study |
| DA49906 | *S.* Emek | *rpoB* S531L | This study |
| DA49908 | *S.* Emek | *rpsL* K42N | This study |
| DA51851 | *S.* Emek | *fusA* P413L | This study |
| DA49912 | *S.* Emek | *fmt* T12R | This study |
| DA49154 | *S.* Emek | *gidB* Q167* | This study |
| DA49188 | *S.* Emek | ∆*mgrB* | This study |
| DA49156 | *S.* Emek | *acrR* H115Y | This study |
| DA49160 | *S.* Emek | *marR* Q110* | This study |
| DA49158 | *S.* Emek | ∆*ompR* | This study |
| DA49190 | *S.* Emek | *lon* Q137* | This study |
| DA50186 | *S.* Emek | ∆*cysB* | This study |
| DA51687 | *S.* Emek | *pmrA* G53E | This study |
| DA9954 | *S.* Indiana | wild type | Strain collection |
| DA49914 | *S.* Indiana | *gyrA* S83F | This study |
| DA49916 | *S.* Indiana | *rpoB* S531L | This study |
| DA49918 | *S.* Indiana | *rpsL* K42N | This study |
| NA | *S.* Indiana | *fusA* P413L | This study |
| DA49922 | *S.* Indiana | *fmt* T12R | This study |
| DA49162 | *S.* Indiana | *gidB* Q167* | This study |
| DA49192 | *S.* Indiana | ∆*mgrB* | This study |
| DA49164 | *S.* Indiana | *acrR* H115Y | This study |
| DA49168 | *S.* Indiana | *marR* Q110* | This study |
| DA49166 | *S.* Indiana | ∆*ompR* | This study |
| DA49194 | *S.* Indiana | *lon* Q137* | This study |
| DA50188 | *S.* Indiana | ∆*cysB* | This study |
| DA51689 | *S.* Indiana | *pmrA* G53E | This study |
| DA49319 | *S. arizonae* | wild type | Strain collection |
| DA49581 | *S. arizonae* | /pPR1347 | This study |
| DA49846 | *S. arizonae* | *gyrA* S83F | This study |
| DA49848 | *S. arizonae* | *rpoB* S531L | This study |
| DA49850 | *S. arizonae* | *rpsL* K42N | This study |
| DA49852 | *S. arizonae* | *fusA* P413L | This study |
| DA49854 | *S. arizonae* | *fmt* T12R | This study |
| DA49651 | *S. arizonae* | *gidB* Q167* | This study |
| DA49649 | *S. arizonae* | ∆*mgrB* | This study |
| DA49653 | *S. arizonae* | *acrR* H115Y | This study |
| DA49655 | *S. arizonae* | *marR* Q110* | This study |
| DA49657 | *S. arizonae* | ∆*ompR* | This study |
| DA49659 | *S. arizonae* | *lon* Q137* | This study |
| DA49661 | *S. arizonae* | ∆*cysB* | This study |
| DA51691 | *S. arizonae* | *pmrA* G53E | This study |
| DA49321 | *S. indica* | wild type | Strain collection |
| DA49585 | *S. indica* | /pPR1347 | This study |
| DA49856 | *S. indica* | *gyrA* S83F | This study |
| DA49858 | *S. indica* | *rpoB* S531L | This study |
| DA49860 | *S. indica* | *rpsL* K42N | This study |
| DA49862 | *S. indica* | *fusA* P413L | This study |
| DA49864 | *S. indica* | *fmt* T12R | This study |
| DA49679 | *S. indica* | *gidB* Q167* | This study |
| DA49677 | *S. indica* | ∆*mgrB* | This study |
| DA49681 | *S. indica* | *acrR* H115Y | This study |
| DA49683 | *S. indica* | *marR* Q110* | This study |
| DA49685 | *S. indica* | ∆*ompR* | This study |
| DA49687 | *S. indica* | *lon* Q137* | This study |
| DA49689 | *S. indica* | ∆*cysB* | This study |
| DA51695 | *S. indica* | *pmrA* G53E | This study |
| DA5438 | *E. coli* MG1655 | wild type | Strain collection |
| DA24100 | *E. coli* MG1655 | /pSIM5-tet | Strain collection |
| DA49824 | *E. coli* MG1655 | *gyrA* S83F | This study |
| DA49826 | *E. coli* MG1655 | *rpoB* S531L | This study |
| DA49828 | *E. coli* MG1655 | *rpsL* K42N | This study |
| DA49830 | *E. coli* MG1655 | *fusA* P413L | This study |
| DA51271 | *E. coli* MG1655 | *fmt* T12R | This study |
| DA47562 | *E. coli* MG1655 | *gidB* Q167* | This study |
| DA46332 | *E. coli* MG1655 | ∆*mgrB* | This study |
| DA47176 | *E. coli* MG1655 | *acrR* H115Y | This study |
| DA47299 | *E. coli* MG1655 | *marR* Q110* | This study |
| DA47208 | *E. coli* MG1655 | ∆*ompR* | This study |
| DA48110 | *E. coli* MG1655 | *lon* Q137* | This study |
| DA48116 | *E. coli* MG1655 | ∆*cysB* | This study |
| DA34465 | *E. coli* MG1655 | *pmrA* G53E | This study |
| DA29723 | *S. typhimurium* LT2 | *rpoB* S531L  *gyrA* S83F | This study |
| DA29725 | *S. typhimurium* LT2 | *rpoB* S531L  *rpsL* K42N | This study |
| DA25264 | *S. typhimurium* LT2 | *rpoB* S531L  *fmt* T12R | This study |
| DA29721 | *S. typhimurium* LT2 | *rpoB* S531L  *fusA* P413L | This study |
| DA25242 | *S. typhimurium* LT2 | *gyrA* S83F  *rpsL* K42N | This study |
| DA48801 | *S. typhimurium* LT2 | *gyrA* S83F  *fmt* T12R | This study |
| DA48800 | *S. typhimurium* LT2 | *gyrA* S83F  *fusA* P413L | This study |
| DA25261 | *S. typhimurium* LT2 | *rpsL* K42N  *fmt* T12R | This study |
| DA48805 | *S. typhimurium* LT2 | *rpsL* K42N  *fusA* P413L | This study |
| DA44662 | *S. typhimurium* LT2 | *rpoB* S531L  *gyrA* S83F  *rpsL* K42N | This study |
| DA46695 | *S. typhimurium* LT2 | *fmt* T12R  *gyrA* S83F  *rpoB* S531L | This study |
| DA25253 | *S. typhimurium* LT2 | *rpoB* S531L  *gyrA* S83F  *fusA* P413L | This study |
| DA46693 | *S. typhimurium* LT2 | *rpoB* S531L  *rpsL* K42N  *fmt* T12R | This study |
| DA52171 | *S. typhimurium* LT2 | *rpoB* S531L  *rpsL* K42N  *fusA* P413L | This study |
| DA44664 | *S. typhimurium* LT2 | *gyrA* S83F  *rpsL* K42N  *fmt* T12R | This study |
| DA25249 | *S. typhimurium* LT2 | *gyrA* S83F  *rpsL* K42N  *fusA* P413L | This study |
| DA46694 | *S. typhimurium* LT2 | *rpoB* S531L  *gyrA* S83F  *rpsL* K42N  *fmt* T12R | This study |
| DA51191 | *S. typhimurium* LT2 | *rpoB* S531L  *gyrA* S83F  *rpsL* K42N  *fusA* P413L | This study |
| DA49284 | *S. typhimurium* LT2 | ∆*ompR*  *marR* Q110* | This study |
| DA49288 | *S. typhimurium* LT2 | ∆*ompR*  ∆*mgrB* | This study |
| DA49286 | *S. typhimurium* LT2 | ∆*ompR*  *lon* Q137* | This study |
| DA49290 | *S. typhimurium* LT2 | *marR* Q110*  ∆*mgrB* | This study |
| DA49292 | *S. typhimurium* LT2 | *marR* Q110*  *lon* Q137* | This study |
| DA49294 | *S. typhimurium* LT2 | ∆*mgrB*  *lon* Q137* | This study |
| DA49345 | *S. typhimurium* LT2 | ∆*ompR*  *marR* Q110*  ∆*mgrB* | This study |
| DA49347 | *S. typhimurium* LT2 | ∆*ompR*  *marR* Q110*  *lon* Q137* | This study |
| DA49349 | *S. typhimurium* LT2 | ∆*ompR*  ∆*mgrB*  *lon* Q137* | This study |
| DA49351 | *S. typhimurium* LT2 | *marR* Q110*  ∆*mgrB*  *lon* Q137* | This study |
| DA51645 | *S. typhimurium* LT2 | ∆*ompR*  *marR* Q110*  ∆*mgrB*  *lon* Q137* | This study |
