# Supplementary material for: Predictable Phenotypes of Antibiotic Resistance Mutations
Source: mBio. 2018 May 15;9(3):e00770-18. doi: 10.1128/mBio.00770-18 (PMC5954217; doi:10.1128/mBio.00770-18)
Supplement: TABLE S4 [file mbo003183881st4.docx]

|  | *S. typhimurium*  LT2 | *S. typhimurium*  14028 | *S. typhimurium*  IVB 5560 | *S. S*aintpaul | *S.* Emek | *S.* Enteritidis | *S.* Indiana | *S. indica* | *S. arizonae* | *E. coli* MG1655 |
| --- | --- | --- | --- | --- | --- | --- | --- | --- | --- | --- |
| *S. typhimurium*  LT2 | 100 | 99.98 | 99.68 | 99.37 | 98.95 | 98.92 | 98.69 | 95.6 | 93.06 | 81.87 |
| *S. typhimurium*  14028 | 1.07 | 100 | 99.67 | 99.35 | 98.98 | 98.94 | 98.7 | 95.6 | 93.07 | 81.86 |
| *S. typhimurium*  IVB 5560 | 0.87 | 0.93 | 100 | 99.4 | 98.98 | 98.95 | 98.68 | 95.6 | 93.12 | 81.83 |
| *S.* Saintpaul | 1.26 | 0.99 | 1 | 100 | 98.99 | 98.95 | 98.72 | 95.61 | 93.12 | 81.75 |
| *S.* Emek | 1.17 | 1.03 | 0.99 | 1.14 | 100 | 99.04 | 98.69 | 95.67 | 93.1 | 81.76 |
| *S.* Enteritidis | 1.33 | 1.3 | 1.24 | 1.35 | 1.39 | 100 | 98.67 | 95.59 | 93.1 | 81.79 |
| *S.* Indiana | 0.21 | 0.9 | 0.99 | 1.05 | 1.06 | 1.32 | 100 | 95.63 | 93.14 | 81.82 |
| *S. indica* | 2.41 | 2.44 | 2.44 | 2.32 | 2.43 | 2.35 | 2.42 | 100 | 92.95 | 81.56 |
| *S. arizonae* | 3.37 | 3.28 | 3.31 | 3.29 | 3.29 | 3.26 | 3.35 | 3.1 | 100 | 81.59 |
| *E. coli*  MG1655 | 5.18 | 4.95 | 4.98 | 5.01 | 4.95 | 5.02 | 5 | 4.92 | 4.75 | 100 |
