# Supplementary material for: Predictable Phenotypes of Antibiotic Resistance Mutations
Source: mBio. 2018 May 15;9(3):e00770-18. doi: 10.1128/mBio.00770-18 (PMC5954217; doi:10.1128/mBio.00770-18)
Supplement: TABLE S5 [file mbo003183881st5.docx]

| **Strain Number** | **Genotype** | **Fitness**  **(SD)** | **Pairwise Epistasis ε** | **Anti-log of epistatic term λ** | **Projected non-epistatic fitness (SD)** |
| --- | --- | --- | --- | --- | --- |
| DA6192 | wild type | 1.00 (0.02) | n/a | n/a | n/a |
| DA28104 | *rpoB* S531L | 0.77 (0.01) | n/a | n/a | n/a |
| DA46691 | *gyrA* S83F | 0.99 (0.02) | n/a | n/a | n/a |
| DA26816 | *rpsL* K42N | 0.78 (0.01) | n/a | n/a | n/a |
| DA46692 | *fmt* T12R | 0.33 (0.01) | n/a | n/a | n/a |
| DA46690 | *fusA* P413L | 0.48 (0.01) | n/a | n/a | n/a |
| DA29723 | *rpoB* S531L  *gyrA* S83F | 0.76 (0.01) | 0.01 | 1.01 | 0.76 (0.02) |
| DA29725 | *rpoB* S531L  *rpsL* K42N | 0.61 (0.01) | 0.01 | 1.02 | 0.60 (0.01) |
| DA25264 | *rpoB* S531L  *fmt* T12R | 0.26 (0.01) | 0.01 | 1.03 | 0.25 (0.01) |
| DA29721 | *rpoB* S531L  *fusA* P413L | 0.44 (0.01) | 0.08 | 1.22 | 0.36 (0.01) |
| DA25242 | *gyrA* S83F  *rpsL* K42N | 0.78 (0.01) | 0.01 | 1.01 | 0.77 (0.02) |
| DA48801 | *gyrA* S83F  *fmt* T12R | 0.33 (0.01) | 0.01 | 1.03 | 0.32 (0.01) |
| DA48800 | *gyrA* S83F  *fusA* P413L | 0.49 (0.02) | 0.02 | 1.04 | 0.47 (0.02) |
| DA25261 | *rpsL* K42N  *fmt* T12R | 0.44 (0.02) | 0.18 | 1.71 | 0.26 (0.01) |
| DA48805 | *rpsL* K42N  *fusA* P413L | 0.40 (0.01) | 0.03 | 1.07 | 0.37 (0.01) |
| DA44662 | *rpoB* S531L  *gyrA* S83F  *rpsL* K42N | 0.63 (0.02) | 0.01 | 1.02 | 0.62 (0.02) |
| DA46695 | *fmt* T12R  *gyrA* S83F  *rpoB* S531L | 0.27 (0.02) | 0.01 | 1.03 | 0.26 (0.01) |
| DA25253 | *rpoB* S531L  *gyrA* S83F  *fusA* P413L | 0.46 (0.01) | 0.00 | 1.00 | 0.46 (0.02) |
| DA46693 | *rpoB* S531L  *rpsL* K42N  *fmt* T12R | 0.30 (0.01) | -0.06 | 0.84 | 0.35 (0.01) |
| DA52171 | *rpoB* S531L  *rpsL* K42N  *fusA* P413L | 0.42 (0.01) | 0.03 | 1.09 | 0.38 (0.01) |
| DA44664 | *gyrA* S83F  *rpsL* K42N  *fmt* T12R | 0.44 (0.02) | -0.01 | 0.97 | 0.45 (0.02) |
| DA25249 | *gyrA* S83F  *rpsL* K42N  *fusA* P413L | 0.39 (0.02) | -0.02 | 0.94 | 0.42 (0.02) |
| DA46694 | *rpoB* S531L  *gyrA* S83F  *rpsL* K42N  *fmt* T12R | 0.27 (0.01) | -0.04 | 0.89 | 0.31 (0.01) |
| DA51191 | *rpoB* S531L  *gyrA* S83F  *rpsL* K42N  *fusA* P413L | 0.41 (0.01) | -0.01 | 0.99 | 0.42 (0.02) |
| DA48717 | ∆*ompR* | 0.99 (0.02) | n/a | n/a | n/a |
| DA48713 | *marR* Q110* | 1.00 (0.02) | n/a | n/a | n/a |
| DA48707 | ∆*mgrB* | 1.00 (0.02) | n/a | n/a | n/a |
| DA48711 | *lon* Q137* | 0.57 (0.05) | n/a | n/a | n/a |
| DA49284 | ∆*ompR*  *marR* Q110* | 0.99 (0.01) | 0.01 | 1.01 | 0.98 (0.03) |
| DA49288 | ∆*ompR*  ∆*mgrB* | 0.99 (0.02) | 0.01 | 1.01 | 0.98 (0.03) |
| DA49286 | ∆*ompR*  *lon* Q137* | 0.67 (0.04) | 0.01 | 1.19 | 0.57 (0.05) |
| DA49290 | *marR* Q110*  ∆*mgrB* | 1.00 (0.02) | 0.08 | 1.01 | 0.99 (0.03) |
| DA49292 | *marR* Q110*  *lon* Q137* | 0.55 (0.06) | 0.01 | 0.96 | 0.57 (0.05) |
| DA49294 | ∆*mgrB*  *lon* Q137* | 0.56 (0.06) | 0.01 | 0.99 | 0.57 (0.05) |
| DA49345 | ∆*ompR*  *marR* Q110*  ∆*mgrB* | 0.98 (0.02) | -0.03 | 0.97 | 1.01 (0.04) |
| DA49347 | ∆*ompR*  *marR* Q110*  *lon* Q137* | 0.68 (0.03) | 0.03 | 1.04 | 0.65 (0.06) |
| DA49349 | ∆*ompR*  ∆*mgrB*  *lon* Q137* | 0.70 (0.03) | 0.03 | 1.04 | 0.67 (0.06) |
| DA49351 | *marR* Q110*  ∆*mgrB*  *lon* Q137* | 0.56 (0.04) | 0.02 | 1.03 | 0.54 (0.05) |
| DA51645 | ∆*ompR*  *marR* Q110*  ∆*mgrB*  *lon* Q137* | 0.70 (0.03) | -0.01 | 0.99 | 0.71 (0.07) |
